# Evaluation of Er Doped CeO_2-δ_ as Oxygen Transport Membrane

**DOI:** 10.3390/membranes12020172

**Published:** 2022-02-01

**Authors:** María Balaguer, Cecilia Solís, Sonia Escolástico, Julio Garcia-Fayos, Jose Manuel Serra

**Affiliations:** 1 Instituto de Tecnología Química, Universitat Politècnica de València-Consejo Superior de Investigaciones Científicas, Avenida de los Naranjos s/n, 46022 Valencia, Spain; soesro@itq.upv.es (S.E.); jugarfa@itq.upv.es (J.G.-F.); jmserra@itq.upv.es (J.M.S.); 2 German Engineering Materials Science Centre (GEMS) at Heinz Maier-Leibnitz Zentrum (MLZ), Helmholtz-Zentrum Hereon, Lichtenbergstr. 1, 85748 Garching, Germany

**Keywords:** ceria, oxygen permeation membrane, energy conversion

## Abstract

Ceria based materials are robust candidates for a range of applications involving redox reactions and high oxygen activity. The substitution of erbium in the ceria lattice introduces extrinsic oxygen vacancies. Further addition of Co introduces electronic carriers. We have studied the structural and redox behavior of Ce_1−x_Er_x_O_2-δ_ (*x* = 0.1 and 0.2) and the influence of adding 2 mol% of Co in the electrochemical properties. A limitation in the solubility of Er cation is found. Diffusion and surface exchange coefficients have been obtained by electrical conductivity relaxation and the DC-conductivity and O_2_ permeation measurements show the importance of the electronic component in the transport properties, obtaining an oxygen permeation flux of 0.07 mL·min^−1^·cm^−2^ at 1000 °C, for a 769 μm thick membrane.

## 1. Introduction

Ceria-based materials lose and adsorb oxygen, shifting from Ce^4+^ to Ce^3+^ in reducing atmosphere and from Ce^3+^ to Ce^4+^ under oxidizing conditions. Charge compensation occurs via oxygen vacancies, which represent the oxygen storage capacity. This redox–activity is suitable for a wide range of applications, e.g., the electrolyte in solid oxide fuel cells (SOFC), oxygen permeating dense membranes, automotive exhaust control, soot oxidation in the automotive industry, catalyst for steam reforming, water-gas shift, hydrogen production, and oxidation reactions [1,2,3,4,5,6].

Ceria-based catalysts show a great benefit for hydrocarbon reforming reactions as they are highly resistant toward carbon deposition and material decomposition by reaction with CO_2_ compared to other conventional metal catalysts and separation materials, as BSCF [7,8]. Therefore, supported ceria membranes have been studied for the oxyfuel process and membrane reactors [9,10].

A complete understanding of the oxygen vacancies formation mechanism is needed for the design of materials focusing on a specific application. Solid-state ion conduction in solids occurs via the transport of oxide ions through a crystal lattice that hosts ionic species of comparable size and opposite charge to the ions. Oxygen ions need to find an empty site in their way and must have enough energy to overwhelm the barrier impeding the diffusion. Thus, the movement of the ions from one to the other side of the material takes place via interstitial sites or by hopping into a vacant site or a complex combination of both interstitial and vacant sites [11]. There are three different ways to create ionic defects in a material (i) intrinsically, by thermal excitation, and extrinsically (ii) by changing the stoichiometry via modifications of the oxygen sublattice, or (iii) by doping with aliovalent cations.

Besides the bulk transport by diffusion, the solid-gas surface exchange of oxygen proceeds via a complex network of surface reaction steps, including chemisorption, dissociation, and incorporation that will influence the overall oxygen transport performance. Therefore, the reaction rates depend on the surface defect concentrations, which are coupled to the bulk concentrations and thus also to the oxygen activity in the gas phase.

Besides, electronic point defects and charge transport play a role in the transport and catalytic properties of doped ceria materials [12,13,14].

The present work focuses on the synthesis of erbium-doped cerium oxide (Ce_1−x_Er_x_O_2-δ_, *x* = 0.1 and 0.2) as a trivalent lanthanide to create extrinsic oxygen vacancies in the ceria lattice and on the influence of adding 2 mol% of Co by impregnation as an electronic aid. We have studied the redox properties of these materials by temperature-programmed reduction (TPR) and oxidation (TPO). Their transport properties in the bulk and surface were studied by DC conductivity and electrical conductivity relaxation (ECR). Finally, dense membranes were tested for O_2_ separation, and the stability in CO_2_ was evaluated.

## 2. Materials and Methods

Erbium-doped ceria materials (Ce_1−x_Er_x_O_2-δ_, *x* = 0.1 and 0.2) were prepared by coprecipitation method. The lanthanide nitrates were dissolved in distilled water at 50 °C. A 1:1.5 molar ratio solution of (NH_4_)_2_CO_3_ was dropped on the lanthanides stirring solution for the complete precipitation of the precursors. The resulting wet powder was rinsed and filtrated and then dried at 100 °C. A solution with the Cobalt nitrate (2 mol% of the doped ceria) was incorporated into the dried precursor powder by incipient wetness impregnation. The fluorite phase was synthesized in a dry air atmosphere at 800 °C for 5 h to decompose the residual nitrates and carbonates.

The crystalline phases were identified by X-ray diffraction by using a PANalytical Empyrean high-resolution powder diffractometer with Mo K_α1,2_ radiation source (λ_1_ = 0.709320 Å and λ_2_ = 0.713600 Å) at the Materials Science Laboratory of Heinz Maier-Leibnitz Zentrum (MLZ). Rietveld refinement, performed by *Fullprof* software [15], has allowed the determination of the lattice parameters and weight fractions. Where possible, the crystallite average sizes and internal strain were obtained by using the instrument resolution file of the diffractometer and taking into account the full-width half-maximum (FWHM) and the shape of the peaks was influenced by the size and internal strain of the particles [16].

Thermogravimetry analysis was performed on a Mettler-Toledo StarE equipment using a heating ramp of 10 °C/min. The gas for decomposing the precursors was air, while the stability was checked in 5% CO_2_ in Ar. Temperature programmed desorption (TPD) measurements were performed by placing 100 mg of powder material sintered at 1200 °C in a quartz reactor and heating it up in the air to 1000 °C, and cooling in the same atmosphere, to eliminate any adsorbed species, as CO_2_ or H_2_O. The oxygen release was monitored in a second step by following the *m*/*z* = 32 and 16 amu, using a He sweep gas and a heating rate of 10 °C·min^−1^ up to 1000 °C, with a mass spectrometer Omnistar (Balzers). Temperature-programmed reduction (TPR) experiments were carried out in a Micromeritics system. A total of 100 mg of powder sample were degassed in Ar for 1 h and later reduced in H_2_/Ar (1/9) at a heating rate of 10 °C·min^−1^ up to 1000 °C. The H_2_ consumption was measured by a TCD.

Rectangular probes (4 × 0.4 × 0.2 cm^3^) of the powders fired at 800 °C were uniaxially pressed at 125 MPa for 1 min and sintered 5 h at 1300 °C in air atmosphere. Electrical conductivity measurements were conducted by standard 4-point DC technique using silver wire and paste for contacting. The measurements were carried out in a temperature range from 800 to 400 °C by a cooling rate of 1 °C·min^−1^ in constant atmospheres under different O_2_ contained atmospheres. The constant current was supplied by a programmable current source (Keithley 2601), and the voltage drop through the sample was detected by a multimeter (Keithley 3706). The conductivity measurements were thermally activated and analyzed on the basis of Arrhenius behavior $\sigma \left(T\right)=\left(A/T\right)exp\left(-E_{a}/kT\right)$. The activation energy *E_a_* was extracted from the slope of the graphs. Once the highest temperature (800 °C) was reached, the samples were left stabilizing for 2 h in each specific *p*O_2_. The electrical conductivity relaxation technique (ECR) was carried out in a plug reactor in which the gas volume was minimized to achieve an almost instantaneous *p*O_2_ change by alternatively swapping oxygen partial pressure from 1 to 0.21 atm at some given temperatures. *D* and *k* were determined by fitting the appropriate solution of Fick’s diffusion equation [17,18] to the experimental electrical conductivity relaxation curves.

Oxygen permeation measurements were performed on a double chamber quartz reactor [19]. Oxygen permeation was studied for Ce_0.8_Er_0.2_O_1-δ_, and Ce_0.8_Er_0.2_O_1-δ_ + Co (2 mol%) gastight membranes consisting of 15 mm diameter discs that were sintered at 1200 °C for 12 h. The thickness of the samples was 769 μm, and the sealing was conducted using gold rings. Oxygen was separated from synthetic air flow using argon as sweep gas. The different feed gas flow rates were controlled using mass flow controllers (MFCs). The permeate gas was analyzed using a micro-GC Varian CP-4900 equipped with Molsieve5A, PoraPlot-Q glass capillary, and CP-Sil modules. Appropriate sealing was confirmed by measuring the nitrogen content in the permeate stream. Reported data were obtained at a steady-state after 30 min of stabilization, and each test was repeated 3 times.

In order to evaluate the stability of Ce_0.8_Er_0.2_O_1-δ_ + Co (2 mol%) membrane under CO_2_-containing atmospheres, 15% CO_2_ in Ar was fed as sweep gas at 750 °C for 24 h. Then, O_2_ permeation was measured sweeping 15% CO_2_ in Ar for 2 different thermal cycles, heating up from 650 °C to 1000 °C and cooling down from 1000 °C to 650 °C.

## 3. Results and Discussion

### 3.1. Synthesis and Structural Characterization

**Figure 1:**
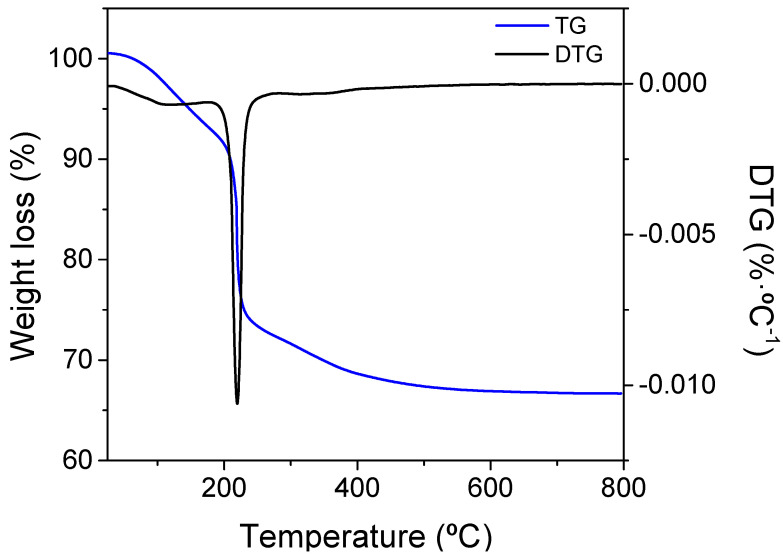
Thermogravimetric and derivative plot of precursor decomposition in the air in the formation of CeEr10.

The formation of the oxide was investigated by monitoring the thermal behavior of the precursors by thermogravimetry. Figure 1 shows DTA/TG curves of the CeEr10 precursor. No clear thermal events corresponding to crystallization processes were identified on the DTA curves. TG curves indicated that the decomposition of CeEr10 precursors into oxides mainly occurred below 400 °C, with a total weight loss of 33%. The Ce^3+^ cations in the nitrates are oxidized to Ce^4+^ during heating, evolving nitrogen oxides. The thermal decomposition of precursors occurs in two steps, (i) the partial decomposition of the ammonium rare-earth carbonates into oxycarbonates and (ii) the decomposition of oxycarbonates into oxides. Therefore, calcination at 800 °C ensures that there will not remain any phase related to the precursor salts, and all the nitrates and (hydroxy)-carbonates disappear completely at this temperature.

**Figure 2:**
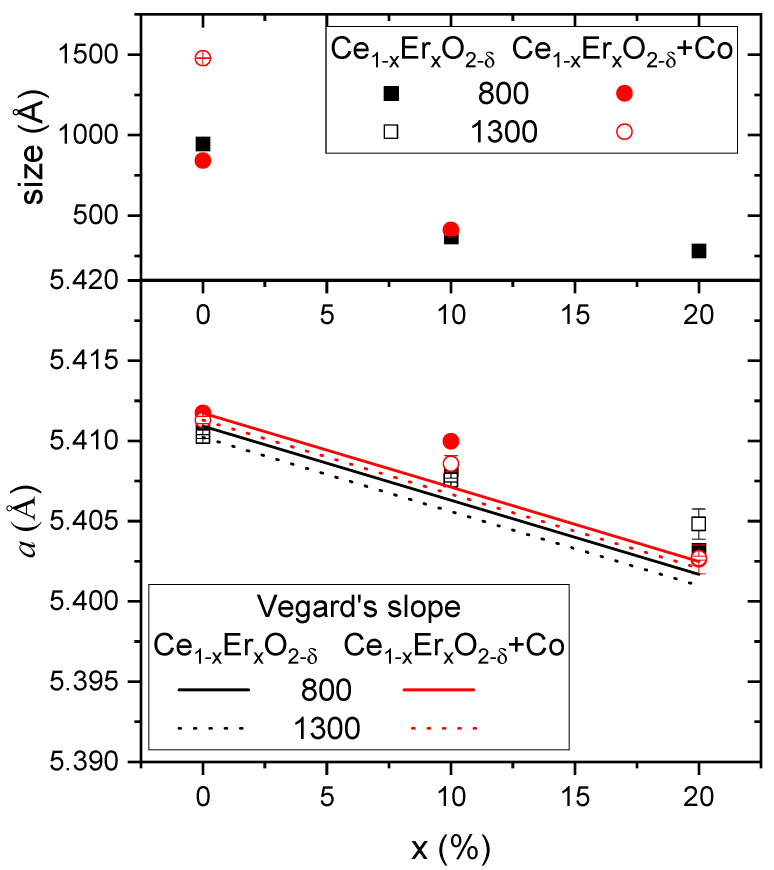
Lattice parameter (**bottom**) and crystallite size (**top**) obtained from Rietveld refinement as a function of the expected Er amount in ceria, *x*. Experimental error is indicated.

The influence of different amounts of Er doping CeO_2_, as well as the sintering temperature and the effect of adding 2 mol% of Co was studied on eight different samples, i.e., Ce_1−x_Er_x_O_2-δ_ and Ce_1−x_Er_x_O_2-δ_ + Co (2 mol%) with *x* = 0.1 and 0.2 and sintered at 800 and 1300 °C. Structural characterization of the obtained materials was carried out by Rietveld refinement of the XRD patterns (Figure S1 and Table S1). All the compounds show cubic fluorite structure with space group $Fm\bar{3}m$. However, and as corresponds to high dopant ratio samples, diffraction peaks corresponding to secondary phases (mainly CoO and Er_2_O_2_) were observed (see supporting Table S1). Co-containing samples show sharper and more intense diffraction peaks due to the grain size growth, highlighting the effect of the well-known effect of Co as a sintering aid [20]. Lattice parameters and crystallite sizes obtained from Rietveld refinement are plotted in Figure 2 as a function of the Er content *x* in %.

The smallest grain size (28 nm) was obtained for the CeEr20 sample sintered at 800 °C. This grain size increased when reducing the amount of Er, reaching 95 nm the CeO_2_ material sintered at 800 °C. Due to the increased crystallization at higher temperature and due to the Co addition, sizes of the samples sintered at 1300 °C with Co were not possible to be obtained by XRD analysis.

Further analysis of the formation of the Er-doped ceria was conducted by the evolution of the cell parameter as a function of the dopant amount. In the absence of secondary phases, the lattice parameter should follow Vegard’s rule, i.e., a linear relationship exists between the cell parameter and the concentration of the solute or dopant [21,22]. Besides, Kim empirical formula provides an estimation of the dopant in ceria by the cell parameter [21,23]:

$$a=a_{0}+\frac{4}{\sqrt{3}}\left[r_{M}-1.024\right]x$$

where $a_{0}$ is the lattice parameter of the corresponding undoped ceria (concerning sintering temperature and Co addition), $r_{M}$ is the ionic radius of the dopant cation and *x* the dopant amount in Ce_1−x_Er_x_O_2-δ_.

**Figure 3:**
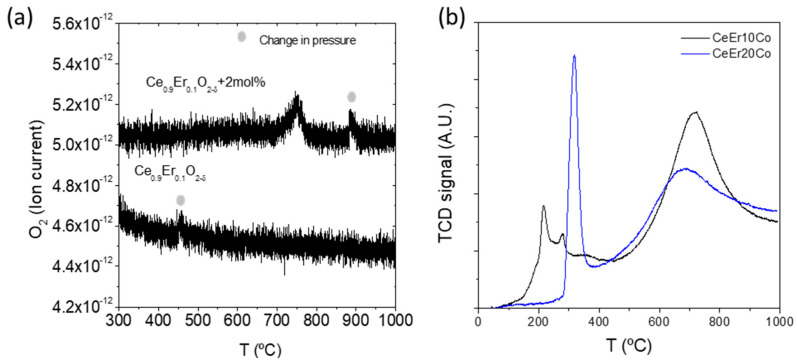
TPD of CeEr10 and CeEr10 + Co (**a**) and TPR of CeEr10 + Co and CeEr20 + Co (**b**) samples.

The difference between the obtained lattice parameters and the expected ones according to Vegard’s slope (also plotted in Figure 3) corresponds to the deviation of the expected amount of Er introduced in the ceria lattice. From the graph, it can be ascertained that the deviation from Vegard’s slope was higher when *x* = 0.1 than when *x* = 0.2, as already found in other studies [24,25]. This can be explained due to the small size of the Er, which results in the possibility of being allocated as interstitial cations. It can be assumed that the plane [1/2.0.0] of the unit cell is the most possible interstitial site for the dopant cation, resulting, as observed, in minimal distortion of the lattice [21,24]. Besides, the assumption of a coordination number less than 8 for cations, due to a non-unique radius of the oxygen vacancy, can produce these deviations [21,24].

### 3.2. Electrochemical Properties

Redox properties of the CeEr10, CeEr10 + Co, and CeEr20 + Co were tested by temperature-programmed desorption and reduction (TPD and TPR), plotted in Figure 3a,b, respectively. The oxygen TPD analysis depicted in Figure 3a shows that only the desorption profile characteristic of Co appears at 760 °C in an inert atmosphere [26,27]. The two small peaks marked in the plot were identified as pressure changes inside the mass spectrometer. The very low reduction will be achieved in inert atmospheres as Ar or N_2_, usually employed as a sweep gas in separation membranes, and the oxygen vacancies are mainly the extrinsic ones provided by the erbium dopant. The TPR profiles (Figure 3b) show several reduction features. The peak at higher temperature corresponds to bulk ceria, indicating that it is only reduced after 400 °C, reaching the maximum reaction rate at 720 and 685 °C for CeEr10 + Co and CeEr20 + Co, respectively. The hydrogen consumption observed for CeEr10 + Co is 14.6 mL·g^−1^, and 10.8 mL·g^−1^. This difference was due to the higher amount of reducible ceria in the first material. The percentage of reduction in the cerium dioxide, nevertheless, will also be affected by the particle dispersion and size and the interaction with CoO_x_ and Er_2_O_3_ particles. The reduction peaks at lower temperatures correspond to surface Co oxides following a two-step reduction, namely from Co_3_O_4_ to CoO and then to metallic Co [28]. The differences in Co reduction pattern are attributed to differences in particle size and distribution of Co, [29] as well as in the erbium content and Er_2_O_3_ in the interstitials. It was shown previously for Ce_1−x_Tb_x_O_2-δ_ (*x* = 0.1, 0.2) + 2 mol%Co that higher ceria content shifts down the reduction temperature of Co as for the present study [26].

**Figure 4:**
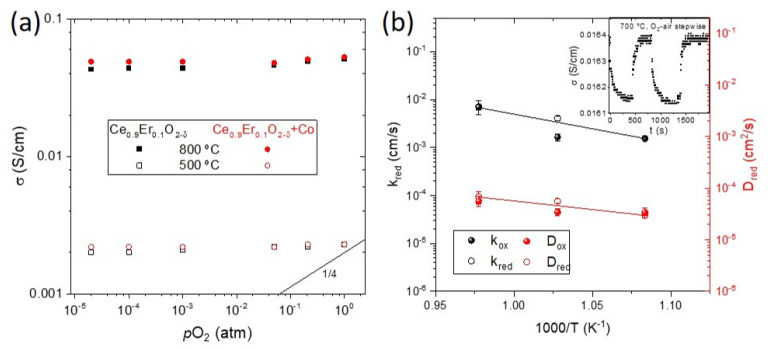
(**a**) Total conductivity at 800 °C as a function of the *p*O_2_ of samples CeEr10, CeEr10 + Co, CeEr20 and CeEr20 + Co sintered at 1300 °C, and (**b**) temperature evolution of the obtained *k* and *D* coefficients of sample CeEr20 + Co_1300, from conductivity relaxation measurements such as the one shown in the inset at 700 °C.

High-temperature transport properties characterization was performed in dense probes sintered at 1300 °C. The transport mechanism was evaluated by DC conductivity measurements performed from RT up to 800 °C in different *p*O_2_ containing atmospheres (see Figure S2). The conductivity dependency on *p*O_2_ at 800 °C is shown in Figure 4a, where also 1/4 and 1/6 dependencies were plotted for comparison with a p-type electronic conductor. In oxidizing atmospheres between 1 and 10^−2^ atm of O_2_, there was a slight decrease of the conductivity with the *p*O_2_, very different from the ¼ expected for *p*-type electronic conductivity. It is attributed to the residual surface reduction of CeO_2_. Overall, the material behaves as an ionic conductor. The detailed mechanism of transport is developed in Supporting Information. Similar conductivity was observed for both dopant levels, which is in accordance with the similar Er percentage substituting ceria in the crystal lattice, estimated by the Kim’s formula, having a similar oxygen vacancy concentration and Er cation interstitials. On the other hand, no clear influence of the electronic carriers was observed for any of the samples, not even for the Co-containing materials, indicating that electronic conductivity, if any, was lower than the ionic contribution in all the cases.

Changes in the nonstoichiometry δ are related to changes in the carrier concentration that influence the total conductivity (σ). This variation can be measured by the electrical conductivity relaxation technique (ECR). After an abrupt change of the oxygen partial pressure, it is possible to estimate the chemical diffusion coefficient (*D*), which characterizes the diffusion kinetics of composition changes and the surface exchange coefficient (*k*) by fitting the transient to Cranck’s equation [26]. The *D* and the *k* of the CeEr10 + Co_1300 were determined by conductivity relaxation curves when changing from pure O_2_ to air atmospheres, and vice versa, at different temperatures, as explained elsewhere [26]. The change in conductivity is very small, as it corresponds to the prevailing ionic character of trivalent doped ceria (the case of Er), and the jump is attributed to the minor electronic contribution promoted by Co incorporation. Note that this electronic contribution was not possible to be unambiguously characterized by the total conductivity measurements, but the small change in conductivity when changing from O_2_-air proves the contribution of the electronic carriers. The obtained values of *k* range from 0.2 to 7.1 × 10^−3^ cm/s and *D* from 0.8 to 6.8 × 10^−5^ cm/s from 600 to 750 °C. These very high values are in the same range to those reported for other doped cerias [26,30]. Both diffusion and surface exchange processes are thermally activated, as can be seen from their Arrhenius behavior shown in Figure 4b, and the activation energies *E_a_* are 2.2 and 0.75 eV for *k* and *D*, respectively. The high *E_a_* of the surface exchange coefficient (*k*) corresponds to half of the electronic bandgap of ceria (*E_g_* CeO_2_ = 5.5 eV) and previously associated with the fact that the rate-limiting step for oxygen surface exchange is the availability of free electronic species required for charge transfer, [31] as expected from the low concentration of electronic carriers, barely limited to the contribution of Co (impregnated at 2 mol%). Saying that a way of improving the performance as a membrane or electrode of this material would be the formation of a composite with an electronic (or mixed) conductor to provide the electronic carriers. It has been successfully shown for other doped ceria, such as CeTb, which even performing lower levels of total conductivity, could form a stable composite with NiFe_2_O_4_ spinel and achieve high levels of oxygen permeation even in harsh atmospheres (containing SO_2_ and CO_2_) [26,32].

### 3.3. Permeation

**Figure 5:**
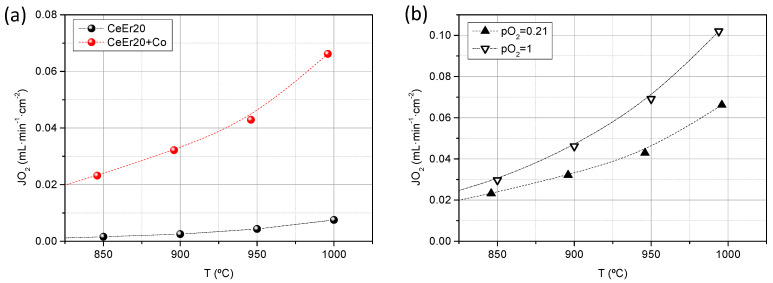
Oxygen permeation flux as a function of the temperature for CeEr20 and CeEr20 + Co sintered at 1200 °C using synthetic air as feed (**a**) and for CeEr20 + Co feeding pure O_2_ and synthetic air (**b**).

In order to experimentally demonstrate the mixed ionic-electronic character of this material and to assess its applicability as O_2_ separation membranes, oxygen permeation tests were performed by using CeEr20 and CeEr20 + Co disk samples of 630 and 769 μm in thickness, respectively, and sintered at 1200 °C. The microstructure of the membranes is displayed in Figure S3, where the higher densification and particle size growth of the samples containing cobalt are confirmed. Figure 5a shows the O_2_ flux for both samples, with 100% selectivity, as a function of temperature using air as the feed gas. CeEr20 presents very low O_2_ fluxes, being almost negligible below 800 °C, ascribed to its very low electronic conductivity. On the contrary, O_2_ flux for CeEr20 + Co reached values of 0.07 mL·min^−1^·cm^−2^ at 1000 °C. At the studied range of temperature, bulk diffusion is the main limiting step as it can be inferred from the activation energy, 0.83 eV (80 kJ·mol^−1^). Therefore, the improvement of the O_2_ flux is due to the higher electronic conductivity introduced by the Co and, subsequently, boosting the ambipolar conductivity. The permeation values are in agreement with similar ceria doped membranes, as can be seen in Table 1. Garcia-Fayos et al. [33] measured a CGO thin film membrane, surface activated with Pd, in which no electronic carriers were introduced. By extrapolating the results to a membrane of thickness similar to this study (769 μm) with the Wagner’s equation, an O_2_ flux of 0.024 mL·min^−1^·cm^−2^ at 1000 °C is estimated. Besides, Balaguer et al. [24] reported the permeation of Ce_0.8_Tb_0.2_O_2-δ_ +Co 2 mol%, measured in the same conditions as in this study, obtaining 0.08 mL·min^−1^·cm^−2^ at 1000 °C, for a 1200 μm thick membrane (0.12 mL·min^−1^·cm^−2^ if extrapolated to 769 μm). These results enlighten the influence of the electronic contribution and nature of the trivalent dopant to the performance of doped-ceria materials as oxygen transport membranes.

The influence of the O_2_ gradient through the membrane was evaluated by pure O_2_ as feed for the CeEr20 + Co sample. As it is observed in Figure 5b, O_2_ flux increases with the *p*O_2_ in the feed, i.e., *p*O_2_ gradient across the membrane, as it is postulated by the Wagner equation [34,35].

**Figure 6:**
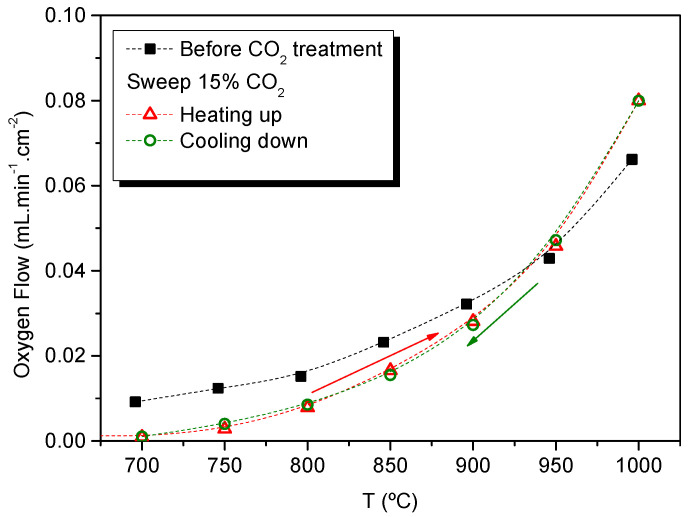
Oxygen permeation flux as a function of the temperature for a CeEr20 + Co sample before CO_2_ stability treatment, and after sweeping 15% CO_2_ in Ar at 750 °C for 24 h heating up from 650 °C to 1000 °C and cooling down from 1000 °C to 650 °C, maintaining CO_2_ in the sweep gas.

One remarkable property of Er-doped cerias is the stability in CO_2_-containing atmospheres (see TG in Supporting Information Figure S4). The influence of O_2_ permeation for Ce_0.8_Er_0.2_O_1-δ_ + Co under CO_2_ atmospheres was evaluated by using 15% CO_2_ in Ar as sweep. The membrane was treated in this atmosphere at 750 °C for 24 h. Afterward, O_2_ permeation was measured heating up from 650 °C to 1000 °C and cooling down from 1000 °C to 650 °C, maintaining the CO_2_ content in the sweep gas. Lower O_2_ fluxes can be observed at temperatures below 950 °C for both thermal cycles (Figure 6). This decrease of the fluxes is ascribed to the CO_2_/O_2_ adsorption competition on the reaction sites rather than the instability of the material [36]. Due to the exothermic nature of the CO_2_ adsorption, this effect is more pronounced as the temperature decreases. On the other hand, O_2_ flux is higher above 950 °C due to the better sweeping properties of CO_2_ as compared with Ar, as has been previously reported [34,36].

## 4. Conclusions

Erbium-doped ceria materials have been studied in order to assess their suitability as an oxygen separation membrane of use at high-temperature applications, as oxyfuel or membrane reactors. XRD and Rietveld refinement examination showed that the substitution of erbium in the ceria lattice is limited, and Er cations may allocate in interstitial sites. The redox behavior shows that only Co is reducible in inert gas conditions. Er doping introduces extrinsic oxygen vacancies at a similar level for 10 and 20% of nominal doping, as observed by DC-conductivity measurements and expected from XRD structural conclusions. Further addition of Co by impregnation introduces electronic carriers, which affect the electrochemical properties. Therefore, ECR and oxygen permeation measurements only provided significant results for Co-containing samples, enlightening the importance of the electronic carriers in the transport properties. The achieved O_2_ permeation flux of 0.07 mL·min^−1^·cm^−2^ at 1000 °C, for a 769 μm thick CeEr20 + Co membrane and the stability in CO_2_ containing atmospheres are in agreement with other doped-ceria membrane materials. Since the main transport limitation is related to the electronic carriers concentration, the combination of these ceria-based materials with a second electronic or MIEC material in a composite may boost the performance of the membrane.

## Figures and Tables

**Table 1:** Reported permeation values for different materials measured in similar conditions.

Material	Permeation (mL·min^−1^·cm^−2^)	Thickness (μm)	Temperature (°C)	Ref.
Ce_0.8_Er_0.2_O_2-__δ_	0.008	630	1000	This work
Ce_0.8_Er_0.2_O_2-__δ_ +Co 2 mol%	0.07	769	1000	This work
Ce_0.8_Tb_0.2_O_2-__δ_ +Co 2 mol%	0.08	1200	1000	[24]
CGO thin film (Pd activated)	0.024	40	1000	[33]

## Data Availability

The data presented in this study are available on request from the corresponding authors.

## Supplementary Materials

The following supporting information can be downloaded at: https://www.mdpi.com/article/10.3390/membranes12020172/s1, Figure S1: Rietveld refinement of different samples, Figure S2: Total conductivity data as a function of 1000/T of the different compounds measured under different oxygen-containing atmospheres. Figure S3: SEM images of CeEr20 and CeEr20+Co membranes surfaced sintered in air at 1200 °C, 12 h, Figure S4: TG patterns of CeEr10 and CeEr20 in a 5% CO_2_/Ar atmosphere and comparison with state-of-the-art material BSCF, Table S1: Lattice parameter, size, microstrain, and other phases present in all the samples.
