# Supplementary material for: Evaluation of Er Doped CeO2-δ as Oxygen Transport Membrane
Source: Membranes (Basel). 2022 Feb 1;12(2):172. doi: 10.3390/membranes12020172 (PMC8877577; doi:10.3390/membranes12020172)
Supplement: Supplementary file 1 [file membranes-12-00172-s001.zip › membranes-1554562-supplementary.pdf]

Supporting Information

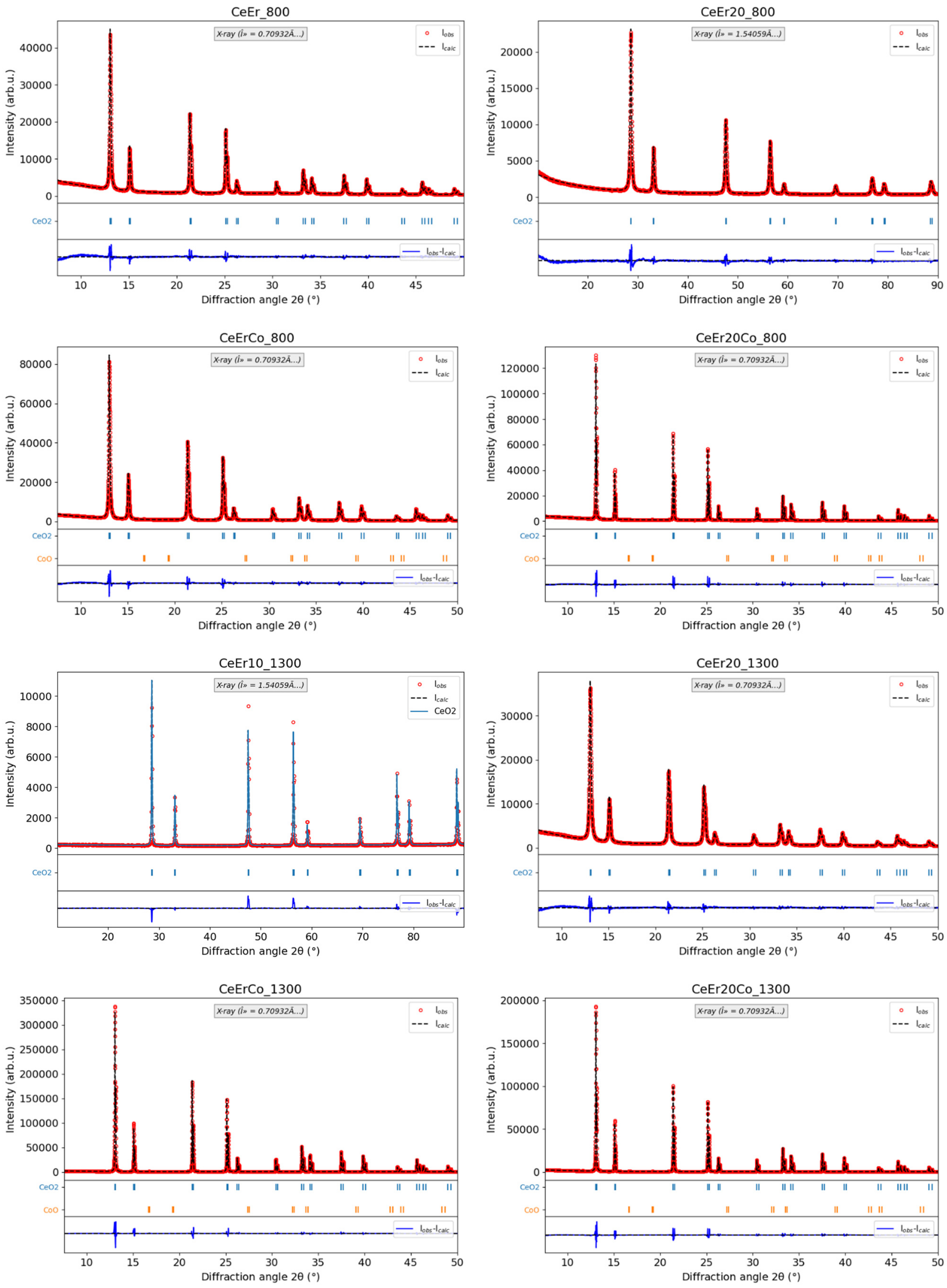

Supporting Figure S1: Rietveld refinement of different Er doped samples

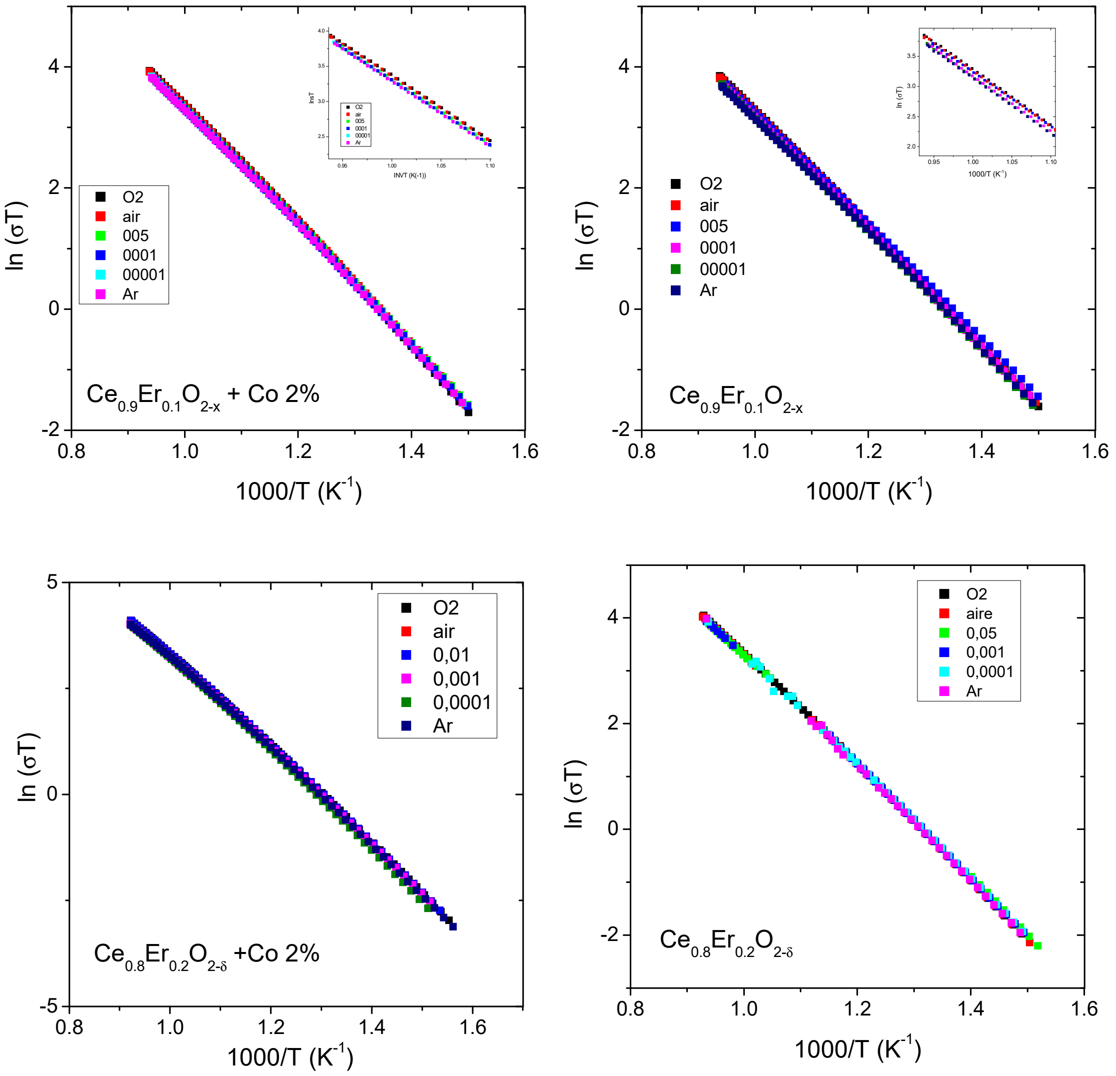

Supporting Figure S2: Total conductivity data as a funciton of 1000/T of the different compounds measured under different oxygen containing atmospheres.

Oxygen Transport Mechanism

The formation of oxygen vacancies and interstitials is dominated by the Frenkel defect mechanism as described in (1) while intrinsic electron-hole pair generation provoked by thermal excitation over the band gap is governed by (2). On the other hand, molecular oxygen release and oxygen vacancy generation (charge compensated by the formation of free electrons localized on Ce ions) and hopping conduction, will occur over the Ce^{3+}/Ce^{4+} from intrinsic defects of pure ceria sublattice following (3).

$$O_O^\times \rightarrow V_O^{\cdot\cdot} + O_i'' ; \quad [V_O^{\cdot\cdot}]\,[O_i''] = K_F(T) \qquad (1)$$

$$nil = e' + h^{\cdot} ; \quad np = K_e(T) \qquad (2)$$

$$O_O^\times \rightarrow \frac{1}{2}O_2\ (g) + V_O^{\cdot\cdot} + 2e' ; \quad [V_O^{\cdot\cdot}]n^2\, pO_2^{1/2} = K_R(T) \qquad (3)$$

In a Er doped system it has to be taken into account the mass conservation law (4), and the added charge defect

$$[Er_{Ce}'] + [Er_i'] = Er_{total} \qquad (4)$$

$$2[O_i''] + n + [Er_{Ce}'] = 2[V_O^{\cdot\cdot}] + p \quad (5)$$

As Er enhances the concentration of oxygen vacancies, the concentration of interstitial oxygen will be suppressed $[O_i''] \rightarrow 0$, improving the ionic conductivity over electronic conductivity in the high pO_2 range, i.e., p → 0 in eq. (5). Besides, in oxidizing conditions and low temperatures most cerium exists as Ce^{4+} and only the extrinsic oxygen vacancies will be present

$$[V_O^{\cdot\cdot}] \propto pO_2^0 \quad (6)$$

Thus, vacancy concentration is independent on pO_2 and proportional to the total erbium concentration in the ceria positions.

Supporting Table S1

Table 1. Lattice parameter, size, microstrain and other phases present in all the samples, number in brackets refers to the error.

**Sample**	***a* (Å)**	**size (Å)**	**strain (%)**	**Other phase**
CeO_2	5.41092(15)	947(2)	1.3656(6)	
CeO_2_1300	5.41027(12)			$Ce_{32}O_{54.24}$?
CeEr10	5.40821(41)	369.3(2)	6.061(4)	
CeEr10_1300	5.40756(14)			0.7% Er_2O_2
CeEr20	5.40319(14)	282.13(12)		0.7% Er_2O_2
CeEr20_1300	5.40548(28)			
CeO_2+Co	5.41174(23)	842(3)		
CeO_2_1300+Co	5.41130(28)	1478		
CeEr10+Co	5.407131(63)	410(1)	8.1328	0.13%CoO
CeEr10_1300+Co	5.408578(58)		4.8(4)	0.2%CoO
CeEr20+Co	5.402614(91)		4.003(1)	1%CoO
CeEr20_1300+Co	5.40269(12)		4.110(1)	0.75%CoO

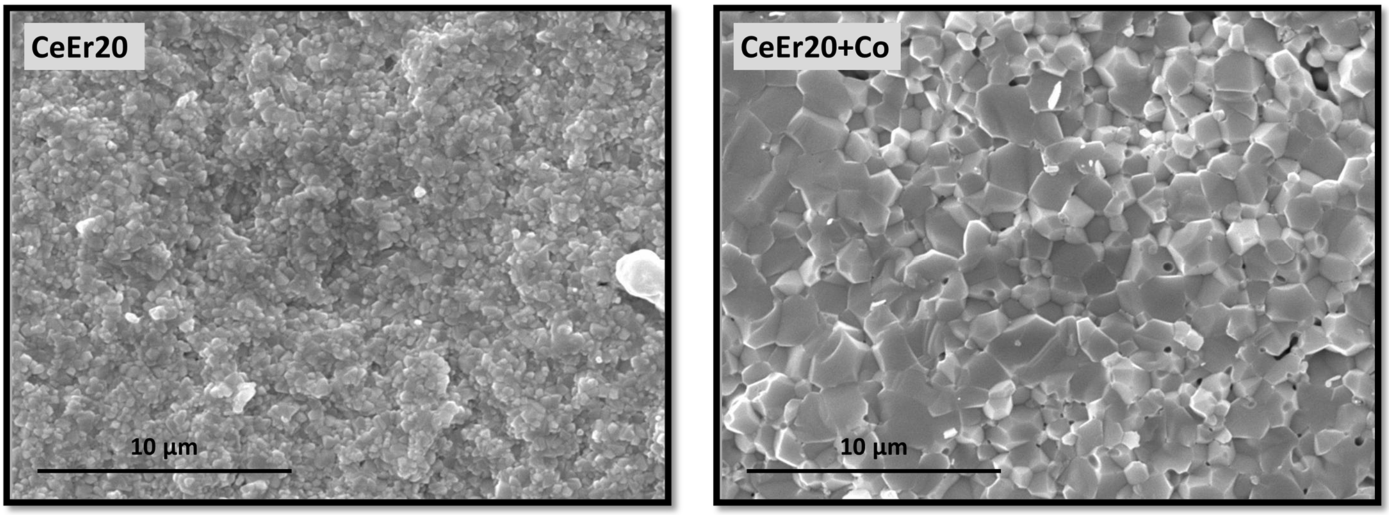

Supporting Figure S3. SEM images of CeEr20 and CeEr20+Co membranes surfaced sintered in air at 1200 °C, 12 h.

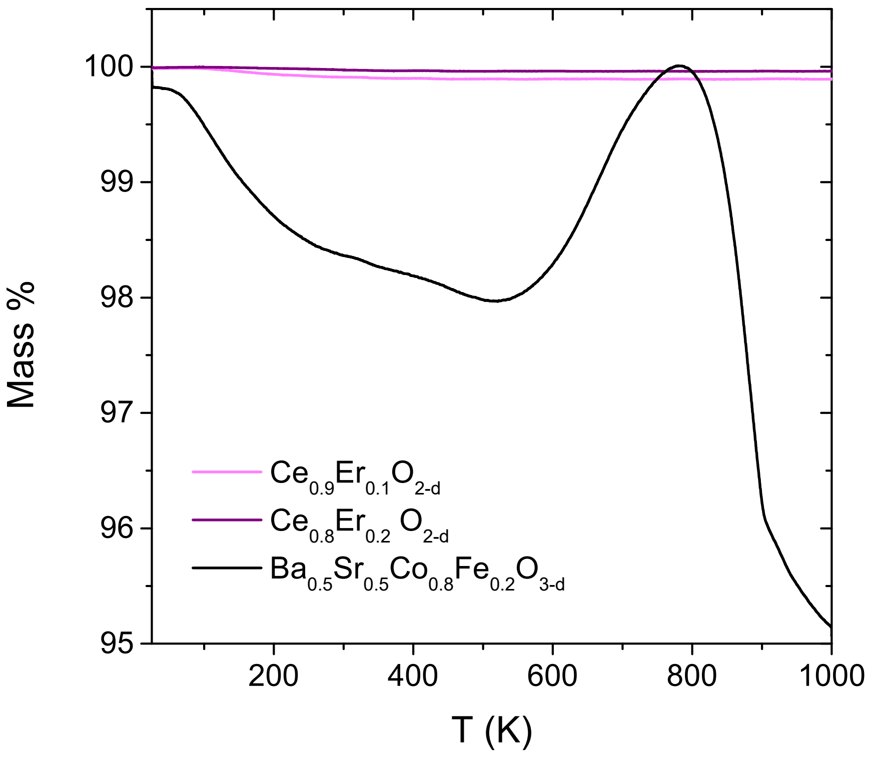

Supporting Figure S4. TG patterns of CeEr10 and CeEr20 in a 5% CO_2/Ar atmosphere and comparison with state-of-the-art material BSCF.
